# Distribution of Mortality

**Published:** 1913-05

**Authors:** 


### Distribution of Mortality

We call special attention to the table of mortality on page 47 of the *Monthly Bulletin of the N. Y. State Dept. of Health.* This table, being as accurate as can be secured and dealing with large masses of population, deserves close analysis by students of vital statistics.

As noted last year, the old fourth reader generalization about the good health and longevity of the country, and the puny, short lived city residents, is no longer justified. For Greater New York the average mortality is 1,427.8 per hundred thousand population. The mortality varies considerably in the different boroughs; is lowest where we should naturally expect, in Queens, but the highest borough mortality is found in Richmond where we should also expect the minimum. Excepting Albany, which has a relatively high mortality (2,016.3) largely because it comes about the nearest of any city of fair size in the United States to having a stationary population, the cities of the second magnitude, in the hundred thousands, average very close to the average for the whole state (1,482.2.) The rural districts, including towns of less than 8,000, have an average mortality of 1,521.2. The small cities show a very great disparity in mortalities; in some cases high on account of large institutions, as for the insane, in some cases phenomenally low, perhaps on account of rapid increase in population by immigration, perhaps due to yearly fluctuations which are neutralized in large communities.

It requires no argument to show that the cities, on account of containing hospitals and institutions, not to mention private practitioners, to whom the most serious cases from the country tend to drift, bear a disproportionate part of the burden of mortality. For example, Lackawanna has the enormous death rate of 699.5 per hundred thousand total population, from diarrhoea-enteritis of children under two, almost ten times the average for the whole state. This is, of course, explained by the noble philanthropy for young children conducted on so large a scale. On the other hand, in spite of very obvious sanitary disadvantages, Lackawanna has a remarkably low typhoid mortality, 6.3 (11.8 for the whole state), or just one case. As a matter of fact, good many typhoid patients from Lackawanna are cared for in Buffalo and some inevitably die. On account of the disproportion in population, such cases markedly reduce the typhoid mortality for Lackawanna without conspicuously increasing that of Buffalo. Yet, in the aggregate, a third or a half of the typhoid cases treated in Buffalo come from outside, and while the mortality from this source is only 11.2, a true mortality of 8 or possibly 5, would give a very different impression and might save heavy expense for engineering.

The variation of tubercular (pulmonary) mortality from 31.3 for North Tonawanda to 314 for the Bronx is noteworthy. Doubtless the fact that the Bronx has double the mortality for tuberculosis, of Manhattan, is due to location of institutions, but the considerable variation of smaller cities, apparently not depending on this cause and certainly not on marked climatic differences may yield to careful investigation, hints as to prophylaxis and treatment.

The marked variations for pneumonia and for infectious diseases aside from tuberculosis, pneumonia and typhoid, are conspicuous for the smaller cities and are doubtless due to yearly fluctuations which are notoriously irregular.

Violent deaths are more common for the country (114.6 as opposed to 99.4 for cities), but vary from 18.6 for Johnstown with a little over 10,000 population to 226.8 for Oneonta, a trifle smaller. Translated into absolute figures of 2 and 23, the probability of chance rather than essential danger is more apparent. In fact congestion of traffic and manufacturing and railroad industries, do not seem to have the bearing on this cause of deaths that one would anticipate.

As already stated, these statistics require careful analysis, to show why the death rate varies so remarkably. Thus analysed, very valuable information may be secured, showing how risk from different modes of death may be avoided.

